# Risk-targeted behavioral activation for the management of work disability associated with comorbid pain and depression: a feasibility study

**DOI:** 10.1186/s40814-022-01040-0

**Published:** 2022-04-23

**Authors:** Michael J. L. Sullivan, Timothy H. Wideman, Nathalie Gauthier, Pascal Thibault, Tamra Ellis, Heather Adams

**Affiliations:** 1 Department of Psychology, McGill University, Montreal, QC H3A 1G1 Canada; 2 School of Physical and Occupational Therapy, McGill University, Montreal, QC Canada; 3 Clinique de Consultation Conjugale et Familiale Poitras-Wright, Coté, Longueuil, QC Canada; 4 Centre for Rehabilitation and Health, Toronto, ON Canada; 5 University Centre for Research and Disability, Halifax, Nova Scotia Canada

**Keywords:** Pain, Depression, Musculoskeletal, Rehabilitation

## Abstract

**Purpose:**

The purpose of the present study was to conduct a preliminary evaluation of the feasibility and impact of a risk-targeted behavioral activation intervention for work-disabled individuals with comorbid pain and depression.

**Methods:**

The design of the study was a single-arm non-randomized trial. The sample consisted of 66 work-disabled individuals with comorbid pain and depression. The treatment program consisted of a 10-week standardized behavioral activation intervention supplemented by techniques to target two psychosocial risk factors for delayed recovery, namely, catastrophic thinking and perceptions of injustice. Measures of pain severity, depression, catastrophic thinking, perceived injustice, and self-reported disability were completed pre-, mid-, and post-treatment. Satisfaction with treatment was assessed at post-treatment. Return to work was assessed at 6-month follow-up.

**Results:**

The drop-out rate was 18%. At treatment termination, 91% of participants indicated that they were “very” or “completely” satisfied with their involvement in the treatment program. Significant reductions in pain (Cohen’s *d* = 0.71), depression (*d* = 0.86), catastrophic thinking (*d* = 1.1), and perceived injustice (*d* = 1.0) were observed through the course of treatment. In multivariate analyses, treatment-related reductions in depression, catastrophic thinking, and perceived injustice, but not pain, contributed significant unique variance to the prediction of return-to-work outcomes.

**Conclusions:**

Risk-targeted behavioral activation was found to be an acceptable and effective intervention for work-disabled individuals with comorbid pain and depression. The findings suggest that interventions targeting psychosocial risk factors for pain and depression might contribute to more positive recovery outcomes in work-disabled individuals with comorbid pain and depression.

**Trial registration:**

ClinicalTrials.gov: NCT0517442. Retrospectively registered.

## Key messages regarding feasibility

- There is currently little evidence about intervention approaches that contribute to occupational re-engagement in work-disabled individuals with comorbid pain and depression.
- The results of this study provide preliminary evidence for the acceptability and effectiveness of risk-targeted behavioral activation (RTBA) as an approach to promoting successful occupational re-engagement in work-disabled individuals with comorbid pain and depression.
- The findings warrant proceeding to a randomized clinical trial to assess the efficacy of RTBA as an approach to fostering occupational re-engagement in work-disabled individuals with comorbid pain and depression.

## Background

Work-related musculoskeletal disorders (WRMD) are a leading cause of disability [1]. In North America, WRMDs rank among the most expensive nonmalignant health conditions affecting the working-age population [1]. Depression has been identified as a risk factor for problematic recovery outcomes of WRMDs [2, 3]. Depressive symptoms contribute to lower productivity and extended periods of sick leave in individuals with WRMDs [3–5]. Depressive symptoms have also been associated with longer duration of salary indemnity benefits following work injury or surgical intervention [6, 7]. Estimates of the prevalence of clinically significant depression associated with work-related musculoskeletal pain range from 20 to 40%, thus making the effective management of disability associated with comorbid pain and depression a pressing clinical concern [8, 9].

Traditional approaches to the clinical management of comorbid musculoskeletal pain and depression have included referrals to mental health services for pharmacotherapeutic or psychotherapeutic treatment or the provision of psychological services within multidisciplinary pain programs [10–13]. While symptomatic treatment is an important component of the clinical management of pain and depression, the results of several investigations suggest that symptom-focused interventions, whether aimed at reducing symptoms of pain or depression, yield only modest reductions in work disability [14–16]. Some symptom-focused interventions, such as the use of opioids for the treatment of persistent pain, have actually been shown to contribute to longer periods of work disability [17]. It has been suggested that reducing disability-relevant risk factors associated with pain and depressive symptoms might lead to more positive return-to-work outcomes than interventions that focus exclusively on symptom reduction [16, 18–20]. To date, research has yet to address the impact of risk-targeted treatment on work-disabled individuals with comorbid pain and depression.

The present study examined the feasibility and impact of a 10-week standardized risk-targeted behavioral activation (RTBA) intervention designed to promote occupational re-engagement in work-disabled individuals with comorbid pain and depression. Behavioral activation is an evidence-based treatment for depression focusing on goal setting, activity scheduling, and problem-solving [21]. The behavioral activation intervention was supplemented with techniques specifically designed to reduce psychosocial risk factors for prolonged work disability, namely catastrophic thinking and perceived injustice. Catastrophic thinking refers to the tendency to anticipate the worst possible outcomes of one’s health or mental health condition [22]. Perceived injustice has been conceptualized as an appraisal process characterized by a tendency to construe one’s losses as severe and irreparable and to attribute blame to others for one’s suffering [23]. High scores on measures of catastrophic thinking and perceived injustice have been shown to prospectively predict more intense symptoms of pain and depression and more prolonged periods of work absence [24–26].

A distinguishing feature of the present study was that occupational reintegration, as opposed to symptom reduction, was the primary objective of treatment. Although research has shown that prolonged work absence is associated with a wide range of adverse health and mental health outcomes, injured workers do not necessarily view occupational resumption as conferring health or mental health benefits [27–30]. Individuals with distressing health or mental conditions readily accept treatments designed to reduce symptom severity; however, they typically do not actively seek out interventions designed to promote occupational reintegration. It is not clear whether an intervention with a stated primary objective of promoting occupational reintegration would be viewed as acceptable by individuals who are absent from work due to comorbid pain and depression.

In addition to examining the feasibility and impact of the RTBA intervention, the present study also addressed whether treatment-related reductions in catastrophic thinking and perceived injustice contribute to more positive return-to-work outcomes. Before advocating for the inclusion of risk-targeted techniques in treatment programs aimed at fostering occupational reintegration, it is necessary to demonstrate that reductions in disability-relevant psychosocial risk factors are associated with more positive return-to-work outcomes. Although previous research has shown relations between disability-relevant psychosocial risk factors and prolonged work absence, there is currently no evidence that reducing catastrophic thinking or perceived injustice fosters better occupational reintegration in individuals with comorbid pain and depression.

The objectives of the present study can be summarized as follows: to determine (1) whether the RTBA intervention was acceptable to participants, (2) whether participants were satisfied with the RTBA intervention, (3) whether the RTBA intervention yielded meaningful reductions in symptom severity and disability-relevant psychosocial risk factors, (4) whether the RTBA intervention yielded meaningful reduction in occupational disability, and (5) to identify the treatment-related changes most predictive of occupational re-engagement.

## Method

### Participants

The initial sample consisted of 66 work-disabled individuals (15 women, 51 men) with musculoskeletal pain and depression. The study sample was recruited through local media advertisements. The media advertisements solicited individuals who had sustained a musculoskeletal injury to the back or neck and were currently absent from work. The inclusion criteria were as follows: (1) work disability greater than 4 weeks associated with a musculoskeletal injury to the back or neck, (2) a score above clinical threshold on a self-report measure of depression, and (3) between 18 and 65 years of age. Exclusion criteria included evidence of any medical condition that would contraindicate participation in an activity-oriented intervention or being scheduled for other medical procedures that would interfere with the participants’ ability to fully engage in the RTBA. Participants who met selection criteria for the study were invited to watch an introductory video that provided detailed information about the procedures (i.e., behavioral activation) and the goals (i.e., return to work) of the RTBA intervention. Participants received $25 for each session of the intervention program they attended. Compensation was provided to defray the costs of inconveniences (e.g., travel, childcare) that might be associated with participation.

#### Risk-targeted behavioral activation (RTBA)

Behavioral activation is a structured intervention that was originally developed for the treatment of depression [21]. Numerous investigations have supported the use of behavioral activation as an effective treatment for depression [31]. There are also indications that behavioral activation might improve return-to-work outcomes in individuals with chronic depression [32]. The behavioral activation component of the treatment program focused on goal setting, structuring and scheduling activities, increasing success and achievement experiences, and problem-solving. In order to maximize the impact of behavioral activation on disability reduction, goal setting and scheduling of activities focused primarily on resumption of *discontinued activities* as opposed to focusing on the scheduling of *pleasant activities*. The objective of goal setting and activity scheduling was to reduce the discrepancy between the client’s pre-injury activity repertoire and the client’s current activity repertoire.

Behavioral activation was supplemented by a collection of techniques designed to reduce catastrophic thinking and perceptions of injustice [20, 26]. Techniques for reducing catastrophic thinking included education, guided disclosure, and increasing behavioral flexibility. Techniques for reducing perceptions of injustice included validation, identifying sources of the client’s perceptions of injustice, and injustice-focused problem-solving. The intervention also involved engaging with the primary care professional, the case manager, and the employer to collaborate on the development of a safe and sustainable return-to-work plan.

Participants first viewed an introductory video that provided an overview of the procedures and objectives of the treatment program. The content of the video clearly identified return to work as a central objective of the treatment program. A client workbook was used for activity scheduling and to guide participants through exercises aimed at reducing catastrophic thinking and perceived injustice.

Treatment was provided in one of 4 participating physical rehabilitation clinics. When a prospective participant was identified, the study coordinator made a referral to the clinic that was in closest proximity to the participant’s community of residence. Clinicians met individually with participants once per week for a total of 10 weeks. The duration of treatment sessions was 50 min. The clinicians for the study included 10 rehabilitation professionals (9 occupational therapists, 1 physiotherapist). All clinicians for the study followed a 2-day training workshop to acquire the skills necessary to deliver the intervention. Clinicians received weekly supervision by a senior clinician with extensive experience in risk-targeted behavioral activation.

### Measures

#### Pain severity

The McGill Pain Questionnaire short form (MPQ-SF) was used to measure pain severity [33]. Participants rated their current pain experience according to 15 pain descriptors on severity scale with the anchors (0) *none*, (1) *mild*, (2) *moderate*, or (3) *severe*. Scores range from 0 to 45 where higher scores represent more severe pain. The MPQ-SF has been shown to be a reliable and valid of pain severity in several different clinical populations [34, 35].

#### Depressive symptoms

The Patient Health Questionnaire-9 (PHQ-9) was used to assess depressive symptom severity. On this measure, respondents indicate how frequently they experience each of 9 symptoms of depression. Ratings are made on a 4-point frequency scale with the endpoints (0) “not at all,” and (3) “everyday.” PHQ-9 scores can range from 0 to 27 with higher scores indicating more severe depressive symptoms. A PHQ-9 score of 10 (i.e., moderate depression) was used as the criterion for clinically significant depression. The reliability and validity of this measure have been established in several different clinical samples [36, 37].

#### Pain catastrophizing

The Pain Catastrophizing Scale (PCS) was used to assess catastrophic thinking related to pain [38]. The PCS consists of 13 items describing different thoughts and feelings that individuals might experience when they are in pain. The PCS has been shown to have high internal consistency (coefficient alpha = 0.87) and to be associated with heightened pain, emotional distress, disability, and employment status [39–41].

#### Perceived injustice

The Injustice Experiences Questionnaire (IEQ) was used to measure injury-related perceptions of injustice [23]. The IEQ consists of 12 items assessing a range of injury-related injustice appraisals. The IEQ has been shown to have high internal consistently and test-retest reliability and to be associated with prolonged occupational disability in individuals with musculoskeletal pain [18, 23].

#### Self-reported disability

The Pain Disability Index (PDI) [42] was used to assess self-rated disability. Respondents rated their level of disability in 7 different areas of daily living (home, social, recreational, occupational, sexual, self-care, life support) due to their pain. The PDI has been shown to be internally reliable and significantly correlated with objective indices of disability [43].

#### Return to work

Return-to-work status was assessed by telephone interview at 6-month follow-up. Participants responded to a number of employment-related questions including whether they had returned to full-time work, part-time work, and transitional work or whether they remained occupationally disabled.

#### Acceptability of treatment

The degree of acceptability of the treatment program was assessed by computing (a) the proportion of respondents who agreed to watch the introductory video, (b) the proportion of respondents who agreed to participate after watching the introductory video, and (c) the proportion of patients who completed the treatment program.

#### Treatment satisfaction

*S*atisfaction with participation in the intervention program was recorded using a 5-point numerical scale with the verbal anchors (0) not at all satisfied, (1) somewhat satisfied, (2) moderately satisfied, (3) very satisfied, and (4) completely satisfied.

### Procedure

This study was approved by Institutional Review Board of McGill University. During an initial telephone screening interview, participants responded to the two questions that comprise the PHQ-2. Participants who scored above clinical threshold on the PHQ-2 during the screening interview, and on the PHQ-9 at the pre-treatment assessment (approximately 2 weeks later), were considered to be experiencing clinically significant symptoms of depression [44, 45]. All participants were enrolled in the treatment program between January 2016 and December 2017. The intervention was offered as a complement to any other pharmacological or physical interventions that might have been prescribed for symptom management. Study measures were completed at pre-, mid-, and post-treatment. Treatment satisfaction was only assessed at post-treatment, and return to work was assessed 6 months following treatment termination.

### Data analytic approach

Descriptive statistics were computed on all study variables. Tests of mean differences were used to compare pre- and post-treatment scores on outcome measures. Percentage change values were computed on measures of symptom severity and psychosocial risk in order to address the clinical significance of treatment-related changes. Correlational analyses were conducted to examine interrelations among indices of change. A logistic regression was used to address the role of treatment-related changes in psychosocial risk measures as determinants of return to work. Tolerance coefficients for all variables included in the logistic regression analysis were greater than 0.60 indicating no problem of multicollinearity. Odds ratios and 95% confidence intervals are presented.

### Sample size

Power analysis was conducted for the logistic regression using power and precision [46]. Assuming medium effect sizes (Cohen’s d) between catastrophic thinking, perceived injustice, and return to work, with power to set at 0.80, a sample of 60 would be required to detect a significant effect (alpha = .05). The assumption of medium effect sizes is supported by research that has been conducted to date [16, 23]. With respect to the objectives of the study, it was considered that an effect size smaller than medium would probably not be of practical significance.

Five participants returned to work prior to completing all 10 sessions of the treatment program. For these participants, mid-treatment assessment results were carried forward and used as their post-treatment evaluation. Since return to work was a treatment objective, participants who returned to work prior to completing all 10 sessions were not considered to have prematurely terminated their involvement in the treatment program. Participants who completed fewer than 10 sessions and did not return to work were considered to have prematurely terminated the intervention (i.e., dropped out).

## Results

### Treatment acceptability

A total of 69 individuals meeting selection criteria for the study responded to the study advertisements. Following a screening interview and having been provided with an explanation of the objectives of the study, 95% (*n* = 66) agreed to watch the introductory video. Of the 66 individuals who agreed to watch the introductory video, all agreed to enroll as participants in the study.

Twelve participants (18%) prematurely terminated the intervention. The main reasons for premature termination included the following: (1) not interested (*n* = 5), (2) accessibility challenges (*n* = 3), (3) another injury or a medical procedure (*n* = 2), and (4) no reason provided (*n* = 2). Of the participants who completed the treatment program (*n* = 54), 91% of participants indicated that they were “very” or “completely” satisfied with their involvement in the program.

### Sample characteristics

Characteristics of the study sample are presented in Table 1. The mean age of the sample was 40.8 years with a range of 25 to 55 years. The majority of participants were married or living common law (80%) and had completed at least 12 years of education (93%). The majority of participants were receiving salary indemnity at the time of enrollment. The sources of salary indemnity were as follows: work injury insurer (30%), disability insurer (45%), motor vehicle insurer (10%), and none (15%). For all participants, musculoskeletal pain was the primary diagnosis for which disability benefits were awarded. The majority of participants (91%) reported that they had received physical therapy treatment at some point since their injury. Concurrent treatments included medication (for pain = 90%, for depression = 45%), physical therapy (30%), occupational therapy (27%), and psychotherapy (20%).

**Table 1:** Sample characteristics

**Variable**	**Men (*N*= 51)**	**Women (*N*= 15)**
Age (years)	41.0 (9.4)	40.2 (8.7)
Education		
Less than high school	6 (12%)	2 (14%)
High school	22 (43%)	6 (44%)
College	14 (27%)	3 (21%)
University	9 (18%)	3 (21%)
Injury site		
Back	40 (78%)	10 (66%)
Neck	11 (22%)	5 (33%)
Occupation		
Labor	19 (37%)	0 (0%)
Trade	11 (23%)	0 (0%)
Health	9 (17%)	6 (40%)
Admin/clerical	5 (9%)	3 (20%)
Sales	3 (6%)	2 (13%)
Food/service	2 (4%)	2 (13%)
Education	2 (4%)	2 (13%)
Duration of work disability		
3–6 months	24 (47%)	5 (33%)
7–12 months	18 (35%)	7 (47%)
More than 12 months	9 (17%)	3 (20%)
	**Mean (SD)**	**Mean**
MPQ-SF (pain)	21.3 (10.5)	22.1 (10.5)
PHQ-9 (depression)	19.0 (7.6)	17.1 (5.4)
PDI (disability)	40.3 (13.4)	38.3 (19.6)

*N* = 66

*PHQ-9* Patient Health Questionnaire-9, *MPQ-SF* McGill Pain Questionnaire-short form, *PDI* Pain Disability Index

Means and standard deviations on the MPQ-SF, PHQ-9, and PDI were similar (within one standard deviation) to those that have been reported in previous research on work-disabled individuals with comorbid pain and depression [47, 48]. Based on scores on the MPQ-SF and the PHQ-9, the study sample would be characterized as experiencing musculoskeletal pain of moderate severity, with moderately severe symptoms of depression at the time of admission.

### Treatment-related changes on dependent measures

*T*-tests for paired samples were conducted to examine treatment-related changes in scores on measures of pain (MPQ-SF), depression (PHQ-9), self-reported disability (PDI), catastrophic thinking (PCS), and perceived injustice (IEQ). Means and standard deviations for assessments conducted at pre- and post-treatment are presented in Table 2. Analyses revealed significant reductions in scores on the MPQ-SF, *t* (53) = 5.8, *p* < .001 (Cohen’s *d* = 0.71; medium effect size), the PHQ-9, *t* (53) = 7.0, *p* < .001 (*d* = 0.86; large effect size), the PDI, *t* (53) = 4.8, *p* < .001 (*d* = 0.95; large effect size), the PCS, *t* (53) = 9.2, *p* < .001 (*d* = 1.1; large effect size), and IEQ, *t* (53) = 8.5, *p* < .001 (*d* = 1.0; large effect size).

**Table 2:** Treatment-related changes in dependent measures

Variable	Pre-treatment	Post-treatment	% change	***d***
**MPQ-SF**	21.5 (9.8)	14.0 (8.4)	−34.0%	0.71
**PHQ-9**	18.5 (7.2)	11.7 (7.3)	−36.0%	0.86
**PDI**	37.0 (15.8)	20.9 (13.2)	−43.0%	0.95
**PCS**	29.6 (9.2)	17.8 (9.9)	−40.0%	1.1
**IEQ**	32.1 (6.5)	24.2 (8.1)	−25.0%	1.0

*N* = 54

*MPQ-SF* McGill Pain Questionnaire short form, *PHQ-9* Patient Health Questionnaire, *PDI* Pain Disability Index, *PCS* Pain Catastrophizing Scale, *IEQ* Injustice Experiences Questionnaire, *d* Cohen’s *d* (effect size)

### Correlations between change scores on risk and symptom measures

As shown in Table 3, reductions in the severity of symptoms of pain (*Δ* MPQ) and depression (*Δ* PHQ9) were significantly intercorrelated (large effect size). Consistent with previous research, reductions in catastrophic thinking (*Δ* PCS) and perceived injustice (*Δ* IEQ) were also correlated with reductions in symptom severity (medium to large effect sizes) [23].

**Table 3:** Correlations between change scores on symptom severity measures and psychosocial risk measures

	Δ MPQ-SF	Δ PHQ-9	Δ PCS
Δ **MPQ-SF**			
Δ **PHQ-9**	0.56**		
Δ **PCS**	0.61**	0.43**	
Δ **IEQ**	0.43**	0.53**	0.67**

*N* = 54

*Δ MPQ-SF =* Change on the McGill Pain Questionnaire short form from pre- to post-treatment, *Δ PHQ-9* = Change on the Patient Health Questionnaire-9 from pre- to post-treatment, *Δ PCS* = Change on the Pain Catastrophizing Scale from pre- to post-treatment, *Δ IEQ* = Change on the Injustice Experiences Questionnaire from pre- to post-treatment

**p < .01

### Return to work

Participants were considered to have successfully returned to work if they were employed full- or part time at 6-month follow-up. On the basis of this criterion, 57% of participants returned to work following participation in the treatment program. The majority (78%) of participants who returned to work returned to their pre-injury employment.

Table 4 shows the results of univariate tests of mean changes on dependent measures as a function of return-to-work outcomes. The results of these analyses revealed that participants who returned to work, compared to participants who remained absent from work, showed significantly greater reductions in pain severity, *t*_Δ MPQ-SF_ (52) = 2.6, *p* = .01, depression, *t*_Δ PHQ-9_ (52) = 2.0, *p* = .05, catastrophic thinking, *t*_Δ PCS_ (52) = 3.6, *p* = .001, and perceived injustice, *t*_Δ IEQ_ (52) = 3.2, *p* = .001.

**Table 4:** Mean differences in change scores on dependent measures as a function of return-to-work outcomes

Return to work				
Variable	No (*n* = 23)	Yes (*n* = 31)	***P***	***d***
Δ **MPQ-SF**	3.0 (9.6)	10.7 (11.4)	.01	0.73
Δ **PHQ-9**	5.4 (6.4)	9.4 (7.4)	.05	0.58
Δ **PCS**	5.6 (11.1)	14.8 (9.5)	.001	0.89
Δ **IEQ**	3.4 (5.0)	10.4 (8.9)	.001	0.96

*N* = 54

*Δ MPQ-SF* = Change on the McGill Pain Questionnaire short form from pre- to post-treatment, *Δ PHQ-9* = Change on the Patient Health Questionnaire-9 from pre- to post-treatment, *Δ PCS* = Change on the Pain Catastrophizing Scale from pre- to post-treatment, *Δ IEQ* = Change on the Injustice Experiences Questionnaire from pre- to post-treatment

Logistic regression was used to examine the value of treatment-related changes in symptom severity and psychosocial risk measures in predicting follow-up occupational status. Age, sex, education, and duration of work absence were entered in the first step as covariates. Change scores for measures of pain, depression, catastrophic thinking, and perceived injustice were entered in the second step of the analysis. Table 5 shows the odds ratios from the final regression equation, *χ*^2^ (8) = 43.2, *p* < .001. As expected, age and education emerged as significant covariates, where older and less educated participants were less likely to return to work. Reductions in depression, catastrophic thinking, and perceived injustice also emerged as significant unique predictors of return to work. Although reductions in pain distinguished between participants who did, and did not, return to work in univariate analysis, reductions in pain did not contribute significantly to the prediction of return to work in the logistic regression.

**Table 5:** Logistic regression examining the value of changes in symptom severity and psychosocial risk measures in predicting occupational re-engagement

Variables	Wald	*df*	Sig.	*OR*	95% *CI*
Age	7.8	1	.01	0.78	0.65–0.92
Sex	1.0	1	0.18	1.07	0.51–13.15
Education	5.1	1	.02	4.09	1.28–11.42
Duration	.06	1	0.95	0.83	0.27–3.99
Δ MPQ-SF	.04	1	0.94	0.99	0.86–1.14
Δ PHQ-9	4.89	1	.03	1.20	1.01–1.42
Δ PCS	5.24	1	.04	1.23	1.03–1.48
Δ IEQ	4.80	1	.02	1.29	1.03–1.64

*N* = 54

Odds ratios and associated significance tests are from the final regression equation

*OR* Odds ratio, *CI* Confidence interval, *Δ MPQ-SF* = Change on the McGill Pain Questionnaire short form from pre- to post-treatment, *Δ PHQ-9* = Change on the Patient Health Questionnaire-9 from pre- to post-treatment, *Δ PCS* = Change on the Pain Catastrophizing Scale from pre- to post-treatment, *Δ IEQ* = Change on the Injustice Experiences Questionnaire from pre- to post-treatment

## Discussion

Feasibility challenges of the present study included participant enrollment and retention in the intervention program. Given that disability reduction is likely seen as a less attractive treatment objective than symptom reduction for individuals with debilitating pain and depression, it was not clear that individuals would volunteer to participate, and fully engage, in the treatment program. The results of the study suggest that it is possible to enroll and engage individuals with debilitating pain and depression in the RTBA intervention. In the present study, of 66 individuals who were invited to participate in the intervention, 90% agreed to view the introductory video, and all individuals who viewed the introductory video agreed to enroll in the intervention program. Only 12 participants (18%) discontinued the intervention program prematurely. Of the participants who completed the intervention, 91% indicated that they were “very” or “completely” satisfied with the treatment they received.

A related feasibility challenge was whether physical therapists and occupational therapists would be able to provide a mental health treatment with the level of proficiency necessary to yield meaningful clinical improvement. It was anticipated that participant retention would be determined, at least to some degree, by participants’ subjective experience of improvement. The results of the study showed that participation in the intervention was associated with clinically significant reductions in pain severity (medium effect size), depression (large effect size), and self-reported disability (large effect size). The effect sizes for reductions in pain severity and self-reported disability were comparable to those that have been reported in work-disabled individuals with chronic musculoskeletal pain participating in pain management interventions [49–51]. The magnitude of reductions in PHQ-9 scores was comparable to that reported in clinical trials of behavioral activation therapy for patients with major depressive disorder [52, 53].

To our knowledge, this is one of only two papers to document the effects of a rehabilitation intervention for individuals with comorbid pain and depression where return to work was a primary outcome variable. At 6-month follow-up evaluation, 57% percent of the study sample had resumed some form of employment. Ernsteen et al. [54] reported 76% of participants with comorbid pain and depression had returned to work after a 57-week multidisciplinary rehabilitation intervention. While yielding more positive return-to-work outcomes than the present study, it is important to consider that the costs associated with a 57-week multidisciplinary intervention would likely be prohibitive for many disability insurers and would likely be beyond the acceptable duration of treatment for many work-disabled individuals. Ernsteen et al. did not report data on acceptability of the rehabilitation program to the target population.

It is useful to compare the return-to-work outcomes of the present study with the return-to-work outcomes of rehabilitation interventions that have been offered to individuals with musculoskeletal pain of a similar level of chronicity, but without comorbid depression. Poulin et al. [55] reported that 55% of participants with persistent pain who completed a 4-week (120 h) multidisciplinary rehabilitation intervention has resumed employment when assessed at 3-year follow-up. Brendbekken et al. [56] reported that 45% of participants with persistent musculoskeletal pain had returned to work when assessed 12 months following completion of a multidisciplinary rehabilitation program. The results of several studies converge to suggest that multidisciplinary rehabilitation for individuals with persistent pain can be expected to yield return-to-work rates of approximately 50% [57]. Given the data showing that depression markedly reduces the effectiveness of rehabilitation interventions for chronic pain, the 57% return-to-work outcome observed in the present study would be considered a favorable outcome for a sample with comorbid pain and depression.

In univariate analyses, reductions in pain severity, depression, catastrophic thinking, and perceived injustice were associated with higher probability of return to work. In multivariate analyses, reductions in depression, catastrophic thinking, and perceived injustice, but not pain, contributed significant unique variance to return-to-work outcomes. Previous studies have also reported that pain reduction predicts return to work in univariate analyses, but not when controlling for pain catastrophizing or perceived injustice [16, 23]. The findings suggest that reduction in pain might influence return-to-work outcomes as a function of its association with pain-related psychosocial risk factors. In previous research, depression, catastrophic thinking, and perceived injustice have been identified as risk factors for delayed recovery in individuals with musculoskeletal pain [24, 58]. The findings of the present study join a growing body of literature suggesting that targeting psychosocial risk factors such as catastrophic thinking and perceived injustice in the treatment of individuals with debilitating health or mental health conditions contributes to reductions in disability, beyond the variance accounted for by treatment-related reductions in symptom severity [16, 59–61].

Strengths of the treatment approach used in the present study was the use of a structured behavioral intervention originally developed for the treatment of depression and inclusion of techniques specifically designed to reduce catastrophic thinking and perceived injustice. Another advantage of the treatment approach used in this study is the low cost of the intervention. RTBA is delivered by one clinician for a maximum of 10 direct contact hours. As such, the cost of treatment (approximately $1.5 k) is only a fraction of the costs associated with multidisciplinary treatment (range $10–35 k). As well, the brief duration of training required to equip clinicians with the skills necessary to deliver RTBA is such that accessibility of the intervention can be readily scaled up to meet the treatment needs of the client population in any given region or jurisdiction. The duration of training required to achieve competency in the delivery of structured behavioral activation interventions is considerably shorter than what would be required to train allied health professionals in the delivery of other psychosocial interventions such as cognitive-behavioral therapy (CBT).

Another advantage of the RTBA intervention used in the present study is that it can be offered as a treatment even when clients are being followed by a psychotherapist. Since the clinicians who delivered the RTBA intervention were not psychotherapists, there would rarely be occasion when the intervention could not be offered in combination with psychotherapy. The orientation of RTBA would be considered conceptually compatible with several psychotherapeutic orientations such as CBT, behavior therapy, and acceptance and commitment therapy. RTBA would be less conceptually compatible with psychotherapeutic orientations that have a more passive or palliative character.

Early detection of depression consequent to musculoskeletal injury remains a challenge due to the service and policy infrastructure within which musculoskeletal injuries are managed. Contact with a health professional able to diagnose depression would be atypical within the pathways of care provided to individuals being treated for work-related musculoskeletal injuries [62, 63]. Assessment of depression is not a routine part of primary medical care for patients presenting with a musculoskeletal injury. As well, insurers are often reluctant to refer clients for psychiatric or psychological consultation due to concerns about increases in claim costs. As a result, depression continues to be under-detected and undertreated in individuals with musculoskeletal conditions [13].

There are several limitations to the present study that invite caution in the interpretation of the study findings. An important limitation was that the study did not include a control group. As well, given the pragmatic nature of the trial, participants continued with treatments that were prescribed by their treating physician. All participants were receiving some form of treatment for pain or depression. Consequently, it is not possible to unambiguously attribute clinical outcomes to the effects of the RTBA intervention. However, most participants who were taking medication for pain or depression had been doing so for a considerable time before entering the trial, making it unlikely that the results could be attributed to the effects of pharmacological treatment. The modest sample size also limited the nature of analytic techniques that could be applied to the data. The present study operationalized mental health comorbidity on the basis of scores on self-report measures as opposed to structured diagnostic interview. Although the bulk of research on depression and pain following occupational injury has been conducted using self-report measures, the diagnostic interview is considered the gold standard for the diagnosis of mental health disorders. Greater confidence in advocating RTBA interventions for the treatment of work disability associated with comorbid pain and depression awaits the results of a randomized controlled trial.

It is also important to consider that the study sample might differ from the population of claimants of injury or disability insurers. Claimants of injury or disability insurers would not be considered “volunteers.” Individuals who volunteer to enroll in a treatment program aimed at fostering occupational re-engagement might present with a higher degree of motivation and engagement than the typical claimant of an injury or disability insurer. This limitation however is not unique to the present study but is relevant to the interpretation and generalizability of all clinical trials using volunteers as participants.

Participants in the present study were offered financial compensation for the involvement in the intervention program. Financial compensation was offered to incentivise participation. While financial compensation for treatment participation would not be characteristic of the conditions under which treatment is typically offered, it is necessary to consider that treatment participation for individuals who are work-disabled due to a musculoskeletal problem is most often incentivized. Treatment referrals for problems contributing to work disability are typically initiated and funded by injury insurers. Included in most injury insurance policies is a clause that requires the claimant to engage in treatment aimed at mitigating losses; failure to do so, particularly when the referral is initiated by the injury insurer, can have adverse claim consequences. Without some mechanism to incentivize participation, few individuals would be enrolling in disability reduction interventions.

Although comorbid pain and depression is considered a very costly health and disability problem, when viewed from a population perspective, work-disabled individuals with comorbid pain and depression are distributed over a wide geographical area. If RTBA was to be considered as a treatment option to managing disability associated with comorbid pain and depression, it would be necessary to develop a plan to disseminate the required skill set to rehabilitation professionals on a region-wide basis. There would be both logistic and financial barriers that would need to be overcome. Mobilizing the resources necessary to train a large and geographically representative group of rehabilitation professionals in the skill set necessary to deliver an RTBA intervention would not be trivial. Furthermore, private sector rehabilitation professionals might not be enthusiastic about relinquishing 2 days of revenue to attend a clinical training workshop that might yield only modest increases in referrals.

One possible solution to this implementation challenge might be establishment of tele-health service delivery units specializing in the delivery of RTBA. Tele-health service delivery would have the advantage of wide geographical reach while requiring a minimal number of trained professionals. The low overhead costs of tele-health service delivery would also keep treatment costs at a minimum. A tele-health service delivery model would also reduce the number of clients who could not enroll or complete the intervention due to accessibility problems. An important future study would be to conduct a clinical trial of the effectiveness of the tele-health delivery of an RTBA intervention.

To summarize, the results of the present study provide preliminary evidence for the acceptability and effectiveness of RTBA as an approach to promoting successful occupational re-engagement in individuals with comorbid pain and depression. The findings further suggest that treatment-related reductions in catastrophic thinking and perceived injustice might be important contributors to successful occupational re-engagement in individuals with comorbid pain and depression. Return-to-work outcomes of individuals with comorbid pain and depression might be enhanced by combining symptom management interventions with interventions specifically designed to reduce catastrophic thinking and perceptions of injustice. The findings warrant proceeding to a randomized clinical trial to assess the efficacy of RTBA as an approach to fostering occupational re-engagement in work-disabled individuals with comorbid pain and depression.
